# Renal Infiltration by Diffuse Large B-Cell Lymphoma as a Rare Cause of Fanconi’s Syndrome: A Case Report

**DOI:** 10.7759/cureus.904

**Published:** 2016-11-30

**Authors:** Shoab Saadat, Syed Nayer Mahmud, Asim Qureshi

**Affiliations:** 1 Department of Nephrology, Shifa International Hospital, Islamabad, Pakistan; 2 Histopathology, Shifa International Hospital, Islamabad, Pakistan

**Keywords:** fanconi’s syndrome, diffuse large b-cell lymphoma, coeliac disease, renal infiltration, hypokalemia, diffuse large b-cell lymphoma (dlbcl), acute kidney injury

## Abstract

We report the case of a 16-year-old female patient with a known history of coeliac disease, who presented with the complaints of diarrhea, vomiting and generalized body weakness. On examination, she was found to have dehydration, decreased power in all her limbs, cervical lymphadenopathy and hepatosplenomegaly. Investigations showed severe hypokalemia, hyponatremia, hypomagnesemia, hypoglycemia and mildly enlarged kidneys on ultrasonography. Biopsy of the duodenum confirmed the flare up of coeliac disease, while cervical lymph node biopsy was positive for atypical lymphoid infiltrate and a morphology suggestive of non-Hodgkin’s lymphoma. The immune profile performed on this sample confirmed the presence of activated/non-germinal center type of diffuse large B cell lymphoma (DLBCL), which was morphologically aggressive in type. The bone marrow biopsy was hypocellular and was negative for any infiltration. The patient was suspected to have developed infiltration of one or both kidneys leading to a rare presentation of Fanconi’s syndrome. She was given first dose of rituximab on the 14th day of her admission. Unfortunately, she developed cardiopulmonary arrest and expired on the next day. We recommend screening for a possible renal involvement in patients with DLBCL and in patients with unusually deranged serum electrolytes as seen in Fanconi’s syndrome. Renal biopsy is considered the gold standard modality for diagnosis and if possible, an earlier sample in a patient with newly developed acute kidney injury can save future complications.

## Introduction

Kidneys are often involved in non-Hodgkin’s lymphoma (NHL) and are reported to be involved in up to 30-40% cases [1]. Most of the times it presents late and is often clinically asymptomatic in the beginning. Clinical presentation in the form of Fanconi’s syndrome is even rarer. We present the case of a young female patient who had a metastasized diffuse large B-cell lymphoma with suspected renal infiltration and clinical presentation mimicking Fanconi’s syndrome. Informed consent was obtained from the patient for this study.

## Case presentation

A 16-year-old female presented on August 25, 2016 to a tertiary care hospital in Islamabad, Pakistan with the complaints of nausea, vomiting, feverish feeling, fatigue and diarrhea. She was a student and belonged to the rural Punjab province of the country. The patient was clinically diagnosed to have coeliac disease and had presented with recurrent episodes of diarrhea and anemia in the past as well. On her several past visits since September 2015 to this hospital, she was found to have low hemoglobin, low albumin, low calcium, low magnesium and low potassium values. She was also tested for HIV in one of her past visits using HIV Ag/Ab combo, which turned out to be negative.

This time her nausea and vomiting had persisted for the previous five days. It contained mostly food particles. There was also a history of several watery, non-bloody diarrheal episodes for three days before the admission, which was associated with feverish feeling and abdominal cramps. The patient had not recorded her temperature and had not taken any medications for it. She also complained of feeling fatigued even on mild exertion. Her fever was of persistent pattern and continued throughout the day. She was received with the above mentioned complaints in the emergency room and was admitted under the medicine services.

Her general physical examination was unremarkable except for the signs of dehydration, fever of 100 degree Fahrenheit and a nodular swelling in the right cervical region. There was also weakness in all her limbs with a power of 3/5 in all the four limbs. There was no sensory deficit or any other positive neurological signs. Her abdominal examination was positive for hepatosplenomegaly. Cardiovascular and respiratory examinations were otherwise normal. Her initial labs showed potassium levels of 3.5 mEq/L, serum iron of 22 mcg/dl and serum TIBC (Total iron binding capacity) of 136 mcg/dl. She was treated along the lines of acute gastroenteritis with intravenous antibiotics and intravenous fluids. The patient had no improvement in her diarrhea and fever, so a duodenal biopsy was carried out on August 27, 2016 to rule out an acute flare up of coeliac disease. A lymph node biopsy was also taken on September 1, 2016 for her unsettling fever.

**Figure 1:**
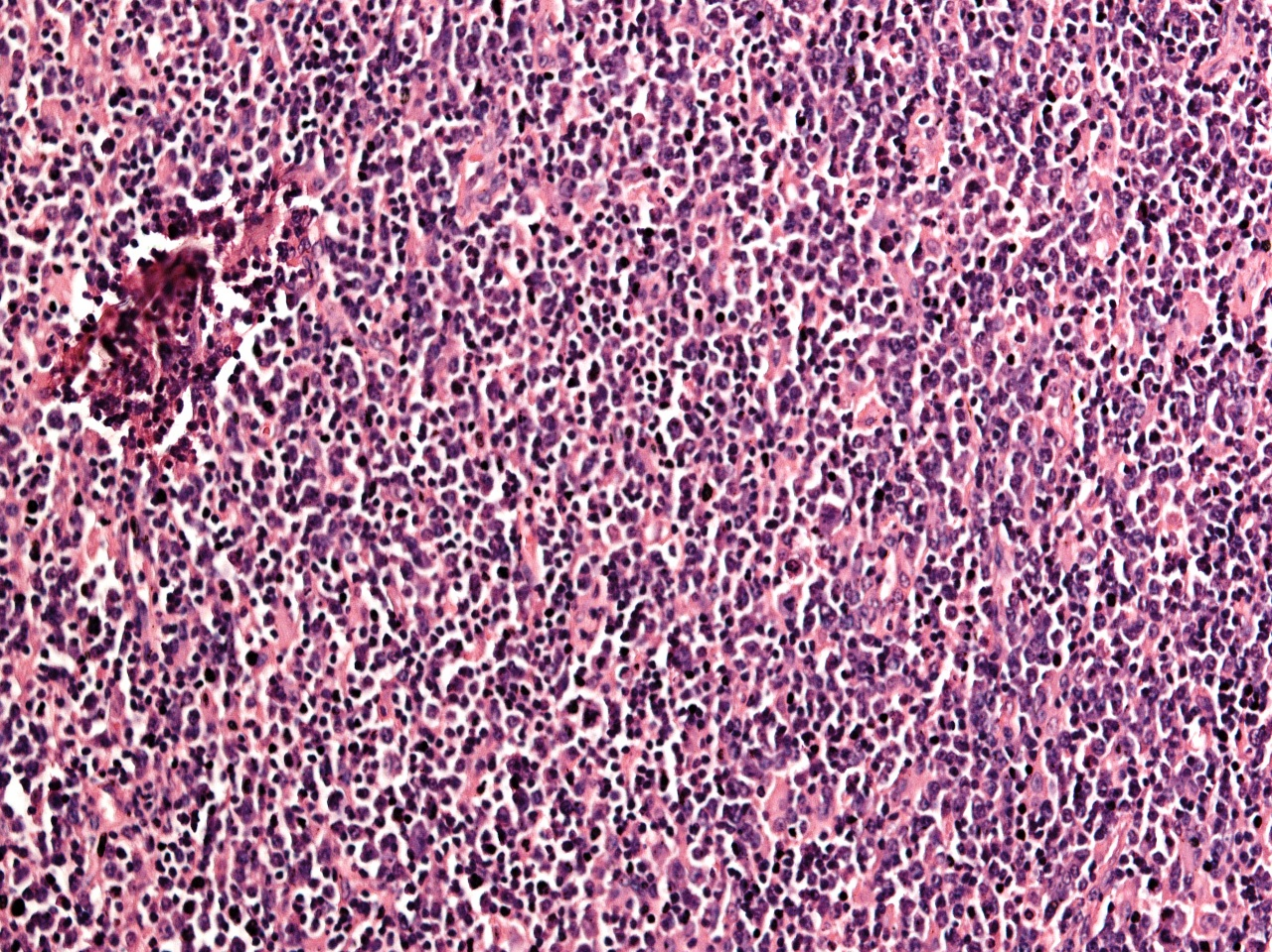
Hematoxyilin (H) and eosin (E) stained slide at 10 X magnification showing diffuse effacement of lymph node by sheets of tumor cells

**Figure 2:**
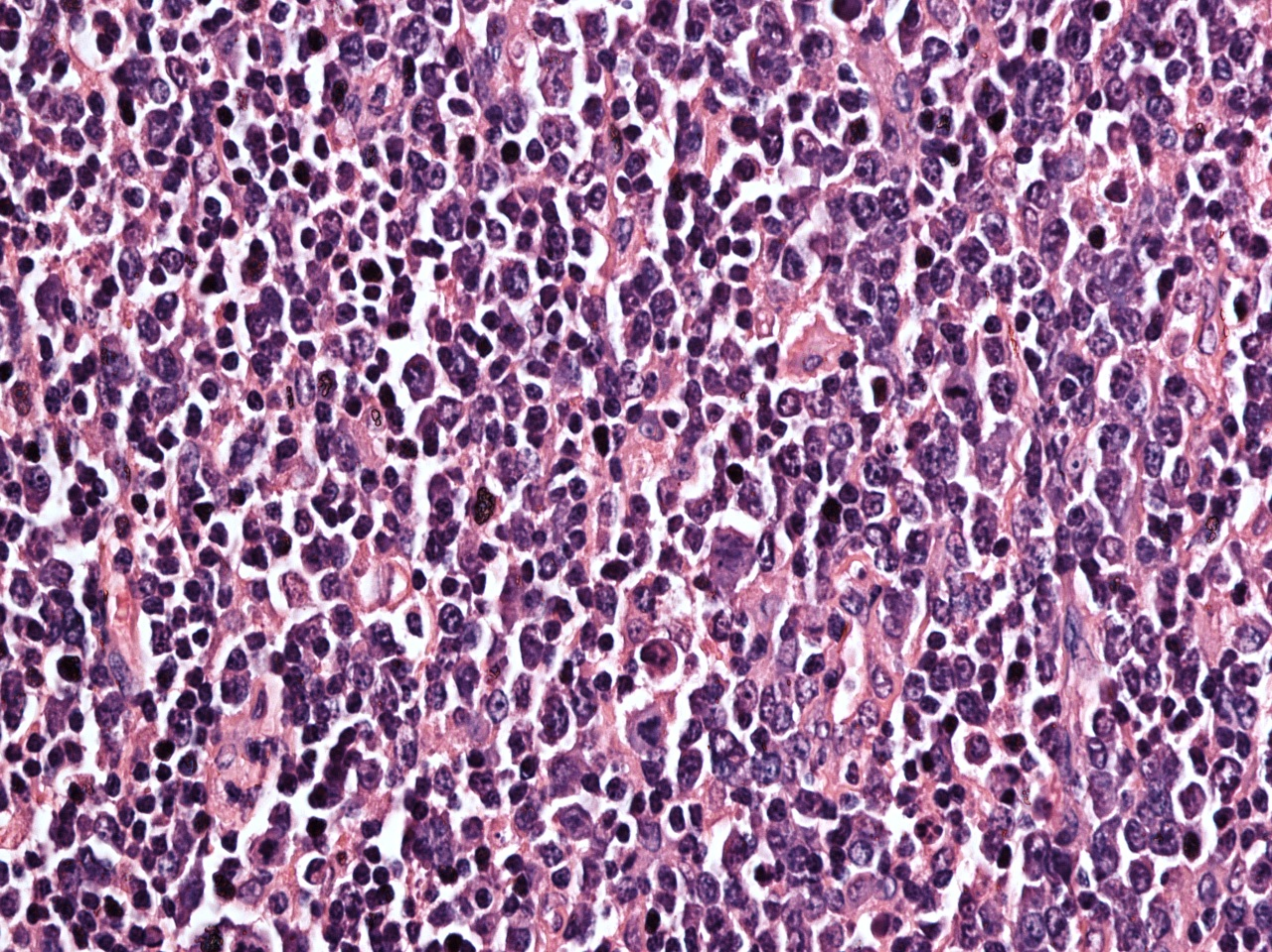
H and E stained slide at 40 X magnification, tumor cells are large with pleomorphic nuclei having prominent nucleoli

**Figure 3:**
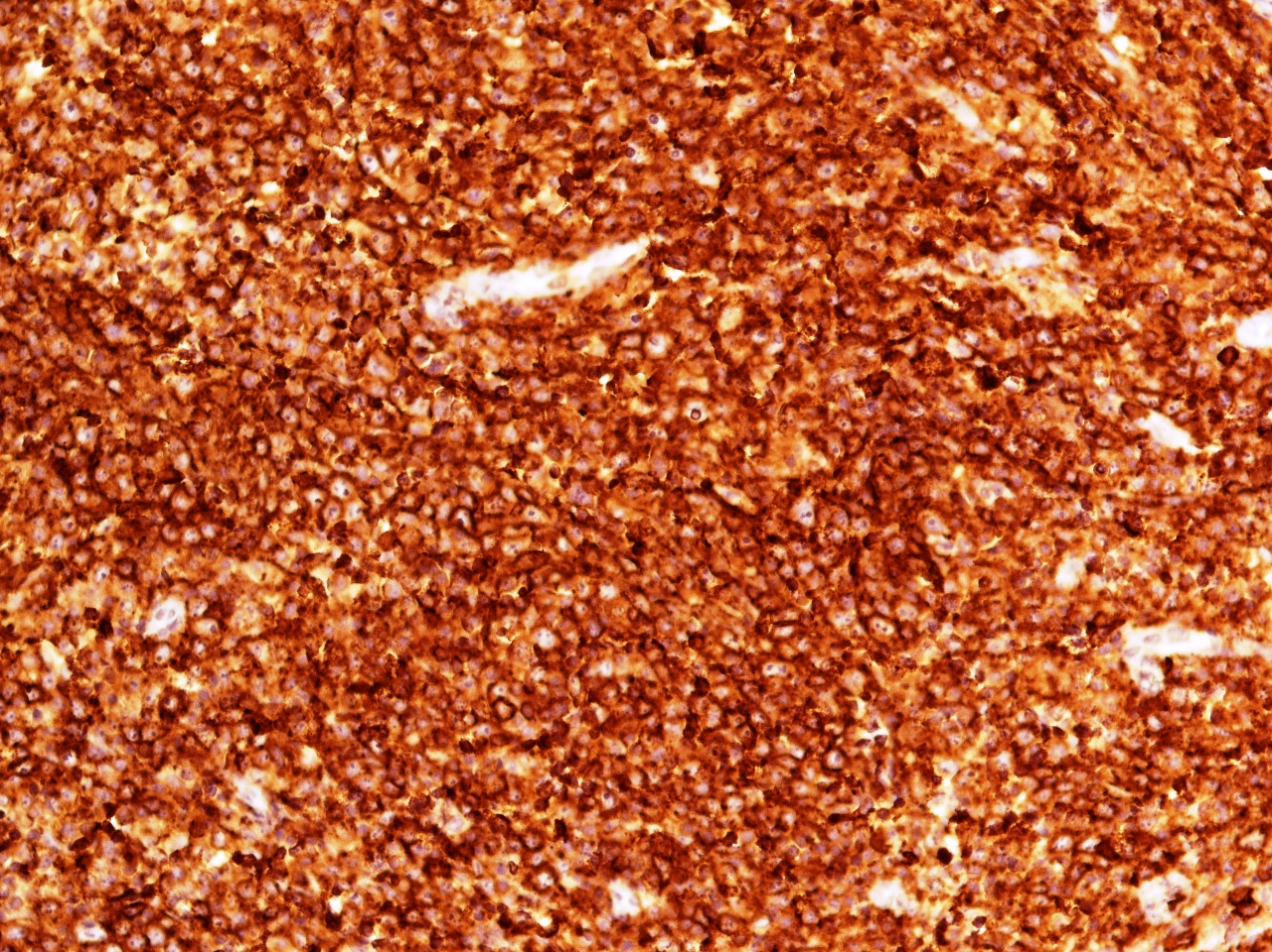
CD20 IHC stain showing diffuse immunoexpression in tumor cells

**Figure 4:**
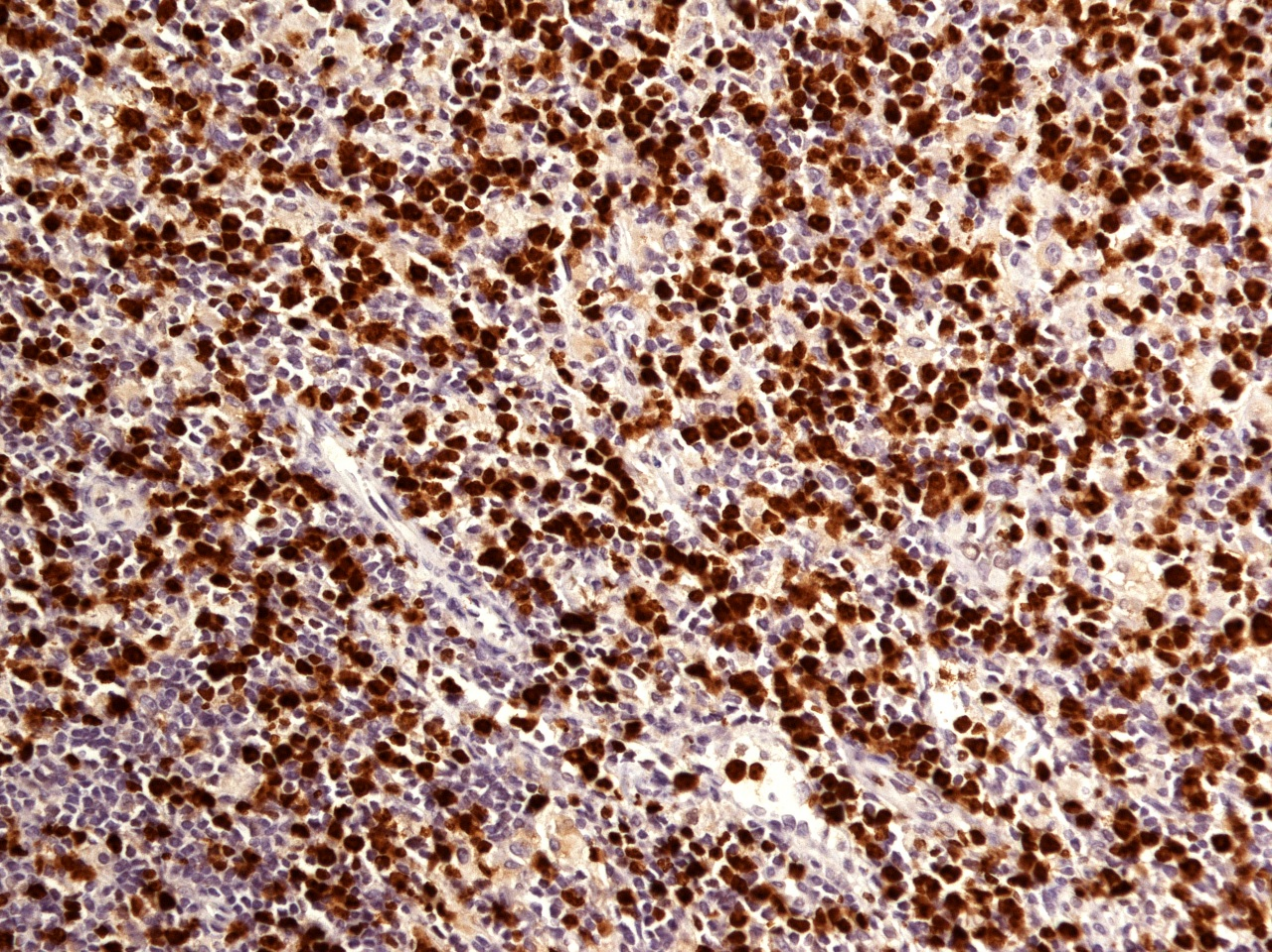
Ki-67 IHC stain shows 60% proliferation rate in tumor cells

Meanwhile, there was also a complaint of burning micturition from the patient for which a urine routine examination (urine R/E) was performed on September 3, 2016, which showed 20-25 white blood cells (WBCs) per high power field, nitrite negative, leukocyte esterase positive and budding yeasts positive as well. A urine culture sent around the same time showed growth the of Candida Albicans > 100,000 CFU/ml, which was sensitive to fluconazole, thus the treatment was started. There was still no improvement in fever after nine days of admission but the diarrhea settled by this time. A liver autoimmune profile was also sent due to a slight rise (aspartate transaminase (AST) =55, alanine transaminase (ALT) =64) in the liver function tests, which showed negative results for antinuclear (ANA), anti-mitochondrial (AMA), anti-smooth muscle (ASMA), anti-gastric parietal cell (GPC), liver kidney microsomal (LKM) antibodies. The duodenal biopsy results arrived early and showed duodenal mucosa with subtotal villous blunting and increased intraepithelial lymphocytes (>30/100 enterocytes). The results from the lymph node biopsy showed complete effacement of nodal architecture (Figure 1). The nodal architecture was completely effaced with sheets of large atypical cells. These cells have pleomorphic nuclei with prominent nucleoli (Figure 2). These cells are diffusely positive for CD20 immunohistochemical (IHC) stain (Figure 3) which is a B cell marker. The proliferation index as determined by Ki-67 was reported as 60% (Figure 4). The morphology and IHC profile was compatible with diffuse large B cell lymphoma. Renal biopsy was not performed in this case but if it was taken, it would also have shown diffuse infiltration of tumor cells.

 

The oncology department was on board and a bone marrow biopsy was performed before the initiation of treatment on September 6, 2016. The results showed a hypocellular marrow with no evidence of bone marrow involvement by lymphoma. It was decided for her to undergo chemotherapy with the first dose of rituximab to be given on September 10, 2016. Her labs on September 9 showed potassium levels of 1.5 mmol/L, serum bicarbonate of 8 mmol/L, magnesium of 1.23 mg/dl, urea of 40.66 and serum creatinine of 1.6 mg/dl, which had risen from a value of 0.79 mg/dl the day before. She had a hemoglobin of 9.30 g/dl. She was started on intermittent intravenous (IV) potassium and magnesium replacement though a central line with regular (every 2 hours) serum potassium measurement. Labs on the next day showed a potassium of 1.3 mmol/L, creatinine of 2.17 mg/dl, serum bicarbonate of 8 mmol/L, magnesium of 2.13 mg/dl. She was put on continuous IV potassium replacement with four-hourly monitoring for serum potassium values.

Meanwhile she underwent the first dose of IV rituximab on floor, which was uneventful. Towards the end of that day the patient started having shortness of breath with decreasing oxygen saturations and a blood pressure reading of 85/40 mmhg. She was immediately shifted to the medical step down (MSD) unit after a rapid response team decided for it. A nephrologist's consultation was asked at this time, who then ordered the continuation of both IV potassium and bicarbonate replacements at 25 mEq per hour. Urine spot creatinine, urine spot potassium and urine spot protein was ordered. Blood gases showed a pH of 6.93, partial pressure of carbondioxide (PCO2) of 27.6 mmhg and PO2 of 103 mmhg. Blood chemistry showed sodium of 135 mmol/L, potassium of 1.3 mmol/L, serum bicarbonate of 8 mmol/L, serum creatinine of 2.17 mg/dl, random serum glucose of 19 mg/dl, anion gap of 31 and magnesium of 2.13 mg/dl. Ultrasonography was done from an outside hospital and showed bilateral mild enlargement of the kidneys. The nephrologist also ordered serum phosphate, uric acid, and depending on the patient’s condition, a renal biopsy. The patient collapsed in MSD and became pulseless. Cardiopulmanary resuscitation (CPR) was started and after 17 minutes of efforts the patient revived. She was intubated and shifted to medical intensive care unit (ICU) for further management. Later, on the same night, she underwent another cardiac arrest and expired. A timeline of laboratory results from the admission till the expiry of the patient is shown in Table 1.

**Table 1:** Timeline of laboratory measurement values

Laboratory measurements	Admission	Day 6	Expiry day (15th)	Reference values
Serum Sodium (mmol/L)	131	128	135	136-145
Serum Potassium (mmol/L)	3.5	3	1.3	3.5-5.0
Serum Bicarbonate (mmol/L)	21	15	8	22-29
Serum Creatinine (mg/dl)	0.5	1.6	2.17	0.6-1.2
Random Blood Sugar (mg/dl)	107	80	19	140-200
Serum Magnesium (mg/dl)	1.53	1.8	2.13	1.7-2.4
Serum Albumin (g/dl)		1.69		3.5-5.5
Hemoglobin (g/dl)	9.9	6.8	9.3	12-15.5
White Cells (per microliter)	11100	10400	8700	4500-11000
Platelet Count (per microliter)	400000	109000	37000	150000-450000

## Discussion

A presentation of a young female patient with a diagnosed coeliac disease, a newly diagnosed diffuse large B-cell lymphoma and a lab presentation mimicking renal proximal convoluted tubular defect (Fanconi’s syndrome) is exceptional. In diffuse large B-cell lymphoma, only 30-40% of the cases have a renal involvement [2]. Our case represents a scenario where there is a suspicion of renal infiltration by this tumor.

The patient in this case report was a known case of coeliac disease. She had presented in the past as well, with episodes of diarrhoea. This time she had a similar presentation but with more pronounced muscular weakness. Also, there was lymphadenopathy and hepatosplenomegaly. Only late into the admission was it realized that there was renal involvement with severe metabolic disturbances. Presence of lymphadenopathy and hepatosplenomegaly suggested an infiltrating disease process. Although, this was unlike some other cases in which there was only bone marrow involvement with no other organ being involved in the disease process [3]. Involvement of kidneys in such a disease process is not rare and the patient may present with painless hematuria, proteinuria or renal failure. Malignant lymphoma can affect kidneys by causing ureteral or vascular obstruction, or by paraneoplastic syndrome leading to hypercalcemia and hyperuricemia. There is also suggestion of glomerulonephritis in a patient with lymphoma [4]. Direct renal parenchymal infiltration leading to acute kidney injury has also been reported in the literature [5]. For a definitive diagnosis of Fanconi’s syndrome, there was indeed a need of renal biopsy and other necessary tests like serum phosphate, serum uric acid, urinary electrolytes, urinary glucose, urine pH and fractional bicarbonate excretion during a bicarbonate infusion. Since the patient expired during the workup, the mentioned tests could not be carried out.

In our patient, development of severe metabolic abnormalities, hyponatremia, hypokalemia, low serum bicarbonate, hypoglycemia, hypomagnesemia, metabolic acidosis and azotemia  suggested possible differential diagnosis like Fanconi’s syndrome, Type 2 renal tubular acidosis, renal injury secondary to medications or urosepsis, glomerulonephritis and post chemo interstitial nephritis. These abnormalities were more appropriately indicative of a proximal convoluted tubule defect (PCT). A suggestive mechanism might have been infiltration of all of these organs, but the development of Fanconi’s syndrome-like presentation as a result of infiltrative process is a rare entity and has been scarcely reported in some case reports around the globe [6]. Fanconi’s syndrome is a transport defect of the proximal convoluted tubule (PCT). It leads to the loss of phosphate, glucose, sodium, potassium and bicarbonate and is associated with metabolic acidosis of type 2 renal tubular acidosis. Mostly it is secondary to an autoimmune disease process like Sjögren’s syndrome, amyloidosis, light chain deposition disease and exposure to heavy metals or other agents, etc. The autoimmune profile of the patient turned out to be negative. Bone marrow and lymph node biopsies were negative for amyloid deposition although a renal biopsy would have been preferred. There was no history of exposure to heavy metals (arsenic, mercury or gold etc) or suggestive of a light chain deposition disease. In our patient, most of the other causes were ruled out for this clinical picture. Since there was an extensive extra-renal involvement, it was postulated that this patient had a secondary metastasis of the kidneys and developed Fanconi’s syndrome as a result of it.

The prognosis of diffuse large B-cell lymphoma with renal involvement leading to Fanconi’s syndrome is variable. An early diagnosis may help in better treatment and control of the disease process. Since there is limited literature available on this rare entity, treatment mostly is the same as for diffuse large B-cell lymphoma alone with necessary fluid and electrolyte replacement for metabolic derangements. A large number of cases show a rapid progression with poor prognosis [7]. Cyclophosphamide, doxorubicin (hydroxydaunomycin), vincristine (Oncovin) and prednisolone (CHOP regimen)  have been the mainstay in diffuse large B-cell lymphoma management [8]. Addition of rituximab to this regimen is shown to improve outcomes [9]. Our patient was initially treated on the lines of acute gastroenteritis and urinary tract infection (UTI). There was also replacement of fluids and electrolytes. After the diagnosis of diffuse large B-cell lymphoma was stablished, she was given the first dose of rituximab on September 9, 2016. She developed a sudden cardiovascular collapse on the night of September 10 and underwent a successful cardio pulmonary resuscitation of 17 minutes at first. Later that night, she was intubated and shifted to critical care unit where she collapsed again and expired.

## Conclusions

In conclusion, Fanconi’s syndrome caused by an infiltrative process of kidneys secondary to diffuse large B-cell lymphoma is a rare entity. To the best of our knowledge, this is the first instance of such a case presentation being presented from Southeast Asia. We suggest that infiltrative lymphoma should be definitely included in the differential diagnosis of a patient presenting with a picture consistent with Fanconi’s syndrome. Also, in a patient with already diagnosed diffuse large B-cell lymphoma, it is suggested to screen for renal involvement at least at the time of presentation and when metabolic derangements are first observed. Renal biopsy is a potent tool and should be carried out along with the use of IHC to help in diagnosing infiltration as the cause of disease process and to guide early therapy.
